# Text-Based Detection of the Risk of Depression

**DOI:** 10.3389/fpsyg.2019.00513

**Published:** 2019-03-18

**Authors:** Jana M. Havigerová, Jiří Haviger, Dalibor Kučera, Petra Hoffmannová

**Affiliations:** ^1^Institute of Psychology, Masaryk University, Brno, Czechia; ^2^Department of Informatics and Quantitative Methods, University of Hradec Králové, Hradec Králové, Czechia; ^3^Department of Pedagogy and Psychology, University of South Bohemia, České Budějovice, Czechia

**Keywords:** depression, genre, morphology, quantitative linguistics, predictive model

## Abstract

This study examines the relationship between language use and psychological characteristics of the communicator. The aim of the study was to find models predicting the depressivity of the writer based on the computational linguistic markers of his/her written text. Respondents’ linguistic fingerprints were traced in four texts of different genres. Depressivity was measured using the Depression, Anxiety and Stress Scale (DASS-21). The research sample (*N* = 172, 83 men, 89 women) was created by quota sampling an adult Czech population. Morphological variables of the texts showing differences (M-W test) between the non-depressive and depressive groups were incorporated into predictive models. Results: Across all participants, the data best fit predictive models of depressivity using morphological characteristics from the informal text “letter from holidays” (Nagelkerke *r*^2^ = 0.526 for men and 0.670 for women). For men, models for the formal texts “cover letter” and “complaint” showed moderate fit with the data (*r*^2^ = 0.479 and 0.435). The constructed models show weak to substantial recall (0.235 – 0.800) and moderate to substantial precision (0.571 – 0.889). Morphological variables appearing in the final models vary. There are no key morphological characteristics suitable for all models or for all genres. The resulting models’ properties demonstrate that they should be suitable for screening individuals at risk of depression and the most suitable genre is informal text (“letter from holidays”).

## Introduction

### Depression

The 10th Revision of International Classification of Diseases ICD-10, which is the basis for diagnosing mental disorders in the Czech Republic, classifies depression as an affective disorder (mood disorder). The disorder can have three forms: mild, moderate and severe forms of depression. One of the first symptoms is a change in mood toward the negative pole: the individual feels sad, needless, and/or unimportant. The disorder significantly affects self-confidence, which is often reflected in social relationships. It is typically accompanied by vegetative symptoms which can manifest as gastrointestinal problems (nausea, diarrhea etc.), tremors, sweating, or dry mouth. Sleep is also affected: the individual may have problems falling asleep, waking up, or staying asleep through the night. The effects of depression extend beyond the individual patient, with negative impact on patients’ employers, spouses, and children (Scott, 1995; Stewart et al., 2003; Sills et al., 2007).

According to the World Health Organization, depression is the most common mental disorder. Currently, 300 million people suffer from depression (WHO, 2017). The prevalence of depression in the adult population (i.e., clinical depression and definite depression) has been reported at approximately 5% across cultures (Molarius and Janson, 2002; Probst et al., 2006; Munce and Stewart, 2007; Romans et al., 2007; Karger, 2014; Klimusová et al., 2016), and approximately 20% in its milder form (partial symptoms, mild depression, and probable depression; Cho et al., 1998; Romans et al., 2007). The same studies also show that depression is almost twice as prevalent among women compared to men (some studies do not confirm these differences, e.g., Angst et al., 2002). The adult group most at risk is the middle-aged population (Murphy et al., 2000; Stordal et al., 2003; Klimusová et al., 2016). Some studies have reported slight differences in prevalence depending on race (e.g., the white population tended to have higher lifetime prevalence than the black population, Somervell et al., 1989) or according to residence (e.g., the prevalence of depression was significantly higher among rural than urban populations at 6.1 versus 5.2%; Probst et al., 2006).

Worldwide, the prevalence of depression in the population is growing, with an increase of 18% between 2005 and 2015 (WHO, 2017). At the same time, early professional intervention results in improvement of psychological symptoms (e.g., lack of self-confidence, rumination, and anticipation of failure) and to elimination of somatic problems (e.g., gastrointestinal problems and sleeping disorders) in 80% of cases (Siu, 2016). Apart from the relief for the individual suffering from depression, intervention and treatment can significantly improve personal and public health as early detection leads to higher chances of returning to personal, social, and economic life (Reavley et al., 2013).

### Goals

Early detection of an individual at risk of depression in initial stages and in mild form is beneficial both for the individual and society. Our study contributes to screening of individuals at risk of this disease. The study provides the original way of depression screening based on an analysis of how the writer uses the language to enable the automatic detection of writer’s risk of depression.

### Related Works

A frequent type of study focusing on the relationship between text variables and mood disorders (e.g., depression) is a case-study. Case studies analyze texts written spontaneously by authors (individuals) suffering from depression. An important example of this approach is Demjén’s (2014) analysis of diaries and works of the American writer Sylvia Plath, who was suffering from lifetime depression and committed suicide in 1963 at the age of 30. In her study, Demjén focused on an analysis of metaphors used by people suffering from depression (i.e., metaphors of separation or loss of control). She found that Sylvia Plath used the second person singular when writing about experiences of conflict or separation. A quantitative analysis of whole texts (not only of the area of metaphors) showed that writers suffering from depression tended to use negative words and expressions with quantifiers with extreme poles (e.g., “everything,” “nothing,” “always,” “never”; Demjén, 2014). Similar results were found in an analysis of texts of the traveler and surveyor Henry Hellyer, who committed suicide at the age of 42. This analysis showed that the pronoun used most frequently was in the first person singular, while the use of the first person plural was much scarcer. Hellyer also tended to use negative words more often, like Sylvia Plath (Baddeley et al., 2011). Pennebaker and Chung (2007) analyzed spontaneous texts of two prominent representatives of Al-Qaeda (Zawahiri and Bin Laden), showing a surprising shift in the use of pronouns closely related to social status, individual and group identity, insecurity, and depression changes.

Automatisation of the processing of linguistic data has recently enabled the use of extensive research strategies. For example, Rude et al. (2004) asked 124 female students attending psychology seminars to write an essay about their deepest thoughts and feelings about college. The students also completed the Beck Depression Inventory, according to which they were divided into groups of currently depressed, formerly depressed and never-depressed people. The authors discovered a positive correlation between degrees of depression and use of the word “I” (i.e., pronouns in the first person singular) and a significantly scarcer use of the pronoun in the second and third persons. It is interesting that other pronouns in the first person singular (“me,” “my” and “mine”) do not show this correlation. Study based on LIWC analyzes conducted by Lieberman and Goldstein (2006) found that women with breast cancer who used more anger words improved in their health and quality of life, whereas women who used more anxiety words experienced increased depression. Ramírez-Esparza et al. (2008) compared the linguistic markers used by people who write about their depression in internet depression forums with linguistic markers used by people with breast cancer on bbc forums in English and in Spanish. It was found that online depressed writers used significantly more 1st person singular pronouns, less first person plural pronouns in both the English and Spanish forums. Women from depressed forums used less positive emotion words and more negative emotion words than women from breast cancer forums in English and Spanish. Sonnenschein et al. (2018) in their LIWC study provide evidence that the texts of people with mood disorders contain increasingly first-person singular pronouns, depressed as well as anxious, but differ in semantic terms (depressed patients used more words related to sadness). Van der Zanden et al. (2014) found that depression improvement during web-based psychological treatments based on textual communication was predicted by increasing use of ‘discrepancy words’ during treatment (e.g., would, should – a conditional in Czech language). Self-referencing verbal behavior appears to have specific interpersonal implications beyond general interpersonal distress and depressive symptoms (Zimmerman et al., 2013). A meta-analysis (*k* = 21, *N* = 3758) of correlations between first person singular pronoun use and individual differences in depression (which occurs in a number of studies dealing with our topic) were conducted by Edwards and Holtzman (2017) who proven evidence that depression is linked to the use of first person singular pronouns (*r* = 0.13), this effect is not moderated by demographic factors, such as gender and there is little to no evidence of publication bias in this literature.

Several studies (e.g., Mairesse et al., 2007; Litvinova et al., 2016b) show that indexes combining several studied markers are also important. For example, a reliable predictor of self-destructive behavior (depression is one of the characteristics of such behavior) is the pronominalisation index: the ratio of pronouns to nouns (Litvinova et al., 2016b).

Existing studies do not only show relationships between the way of writing a text and mood disorders (e.g., depression and associated symptoms), but also a reciprocal healing effect of writing certain types of texts. For example, Sayer et al. (2015) conducted an experiment to test the effects of different writing styles using a sample of 1,292 Afghanistan and Iraq war veterans with self-reported reintegration difficulty. In their experiment, veterans who were instructed to write expressively experienced greater reductions in physical complaints, anger, and distress compared to veterans who were instructed to write factually, and, moreover, both writing groups showed reductions in PTSD symptoms and reintegration difficulty compared to veterans who did not write at all. The correlation between occurrence of words and successful intervention was also documented by Alvarez-Conrad et al. (2001).

Studies in clinical psychology clearly show that research on the relationship between the user of a language (e.g., speaker or writer) and their text is meaningful and has potential for the future. A worldwide and rapidly developing approach is the detection of the personality of authors from their texts, involving the design of predictive models based on correlations between quantifiable text parameters and individual psychological traits (Mairesse et al., 2007; Litvinova et al., 2016b). The present study was designed to add to this body of research.

## The Aim of the Present Research

The study presented here focuses on discovery of the relationship between linguistic characteristics of a written text and the level of the emotional state of depression (depressivity) of its author. The focus is on non-content (non-semantic) computational linguistic markers of a written text. The main objective of the study is to find out which texts (and whether or not) can predict depression and what linguistic characteristics are involved in the eventual model. The key step is to create and evaluate predictive models to detect individuals at risk of depression from written texts. Into the models it is necessary to insert only a limited number of variables (DeVaus, 2002), therefore we carried out a two-stage reduction of input linguistic characteristics: (1) the variables having a low variability will be excluded, (2) the variables that can not distinguish between the depressive and non-depressive respondents will be excluded.

Gender differences: Due to the fact that there are gender differences in depression (Murphy et al., 2000; Stordal et al., 2003; Herring and Paolillo, 2006; Johannsen et al., 2015; Klimusová et al., 2016; Rafi, 2019) as well as gender differences in text processing (Litvinova et al., 2017) our analyses are conducted separately for men and women. We expect the results in each of the samples to differ in some features based on the gender of the writer.

Genre differences: Quantitative linguistic markers of a text are affected by the genre (Douglas, 1992; Stamatatos et al., 2000; Herring and Paolillo, 2006). Thus, the analyses are conducted on texts of four different genres. The genres are divided into two categories: formal (cover letter TXT1 and complaint TXT3) and informal texts (letter from holidays TXT2 and letter of apology TXT4).

Necessity and innovativeness of conducting the present study in Czech: Most research on the relationship between the linguistic properties of a text and its author’s personal traits have been conducted with texts in English (e.g., Pennebaker’s studies above). There is also research on texts in Chinese, Arabic, Spanish, Dutch, French, German, Italian, Russian, Turkish, and Serbian (e.g., Bjekić et al., 2014; Sikos et al., 2014; Sboev et al., 2016). According to Parkvall (2007), Czech is spoken by relatively few native speakers. With 10 million native speakers, Czech is the 83rd most used language in the world and the 15th most frequently used language on the internet. In the studied context, Czech is an under-researched language (W3Techs, 2017) and, with the exception of our own preliminary research, we are aware of no other published research on the relationship between linguistic markers of a text and its writer’s personality in Czech.

## Materials and Methods

### Measures

#### Depression, Anxiety and Stress Scale - 21 Items (DASS-21)

The DASS-21 is a set of three self-report scales designed to measure the emotional states of depression, anxiety and stress. Each of the three subscales contains seven items. Each item is scored on a 4-point scale (0 = *did not apply to me at all*; 3 = *applied to me very much or most of the time*). Thus, a respondent can get 0 to 21 points for each subscale. The DASS-21 is based on a dimensional rather than a categorical conception of psychological disorders. The assumption on which the scale was developed (and which was confirmed by the research data) is that the differences between depression, anxiety and stress experienced by normal subjects and clinical populations are essentially differences of degree (Lovibond and Lovibond, 1995). In this study, we work with the subscale of depression, which assesses dysphoria, hopelessness, devaluation of life, self-deprecation, and lack of interest/involvement, anhedonia, and inertia. In our study, we work with either the total score (0–21 points achieved) or with the cut-off score (non-depressive ≤ 6, depressive > 6), see Lovibond and Lovibond (1995).

*Four fictive letters* were written on a computer in a pre-defined electronic interface. All four letters were written by each participant. The recommended length of texts was 180–200 words. The participants could see the number of words used on the monitor. However, length was recommended, not strictly prescribed. The content of the text could be entirely fictional. The sequence of the four scenarios was selected randomly and each scenario was described to participants as follows:

*Cover letter* (TXT1, formal, positive sentiment): “You have found a job offer that captivated your interest and you aspire to be hired for this position. Therefore, you are going to write a letter to the company’s director as a response to his/her offer trying to persuade the director that you are the right candidate for this position.”

*Letter from holidays* (TXT2, informal, positive sentiment): “You are enjoying your time on an amazing vacation. Everything is going well, as expected, and you fully indulge in your popular activities. Therefore, you have decided to write a letter to your friend and convince him/her to come over and enjoy this perfect time with you.”

*Complaint* (TXT3, formal, negative sentiment): “Until recently, you were satisfied with living in your apartment (or your house), not missing anything. Nevertheless, recent issues have made a hell out of a pleasant living. Although you originally strived to sort out the issues in a polite way, it did not help. Therefore, you decided to write an official complaint to the appropriate authorities.”

*Letter of apology* (TXT4, informal, negative sentiment): “You have done something that substantially harmed your relationship with a person you were very close to for a long time. You had promised something that you did not fulfill. You feel sorry and you know that you made a mistake. Because you do not want to lose your close friend, you have decided to write a letter of apology to him/her.”

The analyses were conducted on 688 texts that create a corpus of 99,481 words. In all texts, quantitative linguistic variables on various levels of classification (e.g., number of all adjectives, number of superlative forms of adjectives, number of words in singular, etc.) were automatically detected in the process of lemmatization with morphological tagging (Jelínek and Petkevič, 2011).

Quantitative linguistic variables are included in the analyses in the form of relativized isolated features (ratios) and compound indicators (special metrics) as described in the following lists.

Ratios (input = 16 items):

– words per sentence: the number of words divided by the number of sentences,
– lemmas per sqrt words: the number of different lemmas (basic forms) divided by the square root of the number of words,
– sentence complexity: the number of finite verbs divided by the number of sentences,
– punctuations per sentence: the number of punctuation marks divided by the number of sentences,
– exclamation per sentence: the number of exclamation marks divided by the number of sentences,
– AN per ANNA: the number of adjective-noun pairs divided by the number of all pairs (adjective-noun plus noun-adjective),
– colloquial words per sentence: the number of colloquial words divided by the number of sentences,
– singularity index: the number of words in singular divided by the number of all words which have the grammatical category of interest (i.e., divided by the number of singular plus the number of plural plus the number of dual nouns),
– singularity P index: the number of possessive singular words divided by the number of all possessive words,
– vocative index: the ratio of words in vocative to the sum of all other words that have the grammatical category of interest,
– negativity index: the ratio of negative sentences to negative plus affirmative sentences,
– passive index: the number of words in passive divided by the number of words in passive and active,
– imperfectum index: the ratio of perfectum to perfectum plus imperfectum,
– dem per words: the number of diminutive words divided by the number of all words,
– vul per words: the number of vulgarisms divided by the number of all words,
– clq per words: the number of colloquial words divided by the number of all words.

Special metrics (input = 8 items):

– coherence index: calculated using the formula Coh = (particles + conjunctions + prepositions)/(3 ^∗^ sentence) (Litvinova et al., 2016b),
– pronominalisation index: the ratio of the total number of pronouns to the total number of nouns (Litvinova et al., 2016b),
– formality metric: is calculated using the formula F = (noun + adjective + preposition + article - pronoun - verb – adverb - interjection + 100)/2 (Mairesse et al., 2007),
– trager index: number of verbs/number of adjectives (Sboev et al., 2016),
– readiness to action: number of verbs/number of nouns (Sboev et al., 2016),
– aggressiveness index: number of verbs/number of all words (Sboev et al., 2016),
– activity index: number of verbs / (number of verb + adjective + adverbs),
– autosemantic index: number of autosemantic words (noun, adjective, pronoun, numeral, verb, and adverb) in relation to number of words (Čech et al., 2014).

### Data Collection Procedure

Participants were recruited using leaflets and advertisements on social networks. The participants were couples of people older than 15 who enrolled in the study voluntarily. After the study, they were awarded about 50 USD. Data collection was conducted in the controlled environment of a university on weekends from September 2016 to April 2017.

A battery of self-report psychological tests was administered with 4 fictive letters placed randomly between test blocks. The conditions of administration were always identical (the same environment, the same assistants) and relatively naturalistic to make the participants feel comfortable (they were allowed to relax when needed and an assistant was present). The maximum level of structure and identity of situation were strictly obeyed to eliminate the impact of structure of the situation on the correlation between linguistic markers of a text and its writer’s personality (e.g., as discussed by Hirsh and Peterson, 2009).

### Participants

Quota selection was used to sample participants. The decisive criterion for determination of quotas was age, gender, and education (Škrabal, 2014). The inclusion criteria were Czech citizenship, command of Czech as mother tongue, good psychical condition (without medication with psychopharmaceuticals), good knowledge of each other in each enrolled couple (that would allow the participants to describe each other sincerely and with a detached view). The participants declared fulfillment of conditions by signing a detailed informed agreement.

The sample is made of *N*_resp_ = 172 respondents, out of whom *n*_m_ = 83 men, *n*_w_ = 89 women. The distribution with respect to age and education is given in Table 1. The studied properties of the research sample correspond to distribution of the legally competent population in the Czech Republic, which makes generalization of results at this level possible.

**Table 1:** Age group, education, and gender of participants (*N*_resp_ = 172).

			% of Total			
			Education			Total %
Gender			Primary %	High school %	University %	
Male	Age group	Younger than 25	7.2	7.2	1.2	15.7
		25–34	1.2	14.5	6.0	21.7
		35–55	3.6	30.1	8.4	42.2
		Older than 55	2.4	12.0	6.0	20.5
	Total		14.5	63.9	21.7	100.0
Female	Age group	Younger than 25	5.6	5.6	1.1	12.4
		25 to 34	1.1	11.2	5.6	18.0
		35 to 55	2.2	24.7	6.7	33.7
		Older than 55	2.2	27.0	6.7	36.0
	Total		11.2	68.5	20.2	100.0
Total	Age group	Younger than 25	6.4	6.4	1.2	14.0
		25 to 34	1.2	12.8	5.8	19.8
		35 to 55	2.9	27.3	7.6	37.8
		Older than 55	2.3	19.8	6.4	28.5
	Total		12.8	66.3	20.9	100.0


### Data Analysis

The analysis was conducted in seven steps. *Outlier filtering*: All texts showing outlying values for number of words (<100) and number of sentences (<5) were discarded. *Reduction of variables, step one: low variability variables exclusion*. Descriptive statistics for the studied QL variables were calculated and variables showing low level of variability were discarded from further calculations (if at least one of the following conditions were fulfilled: mdn = 0 or coefficient of variation sd/m < 0.05 or iqr/mdn < 0.05). *Assessment of normality*: A Kolmogorov–Smirnov (K–S) test was conducted. *Lowering granularity of depressive scale*: The depression subscale of the DASS-21 (min–max = 0–21) was transformed to DASS21_01 (non-depressive ≤ 6, depressive > 6), see Lovibond and Lovibond (1995).

*Reduction of variables, step two: exclusion variables with non-significant intergroup differences* (depressive vs. non-depressive). This comparison was done using the Mann–Whitney test. It was conducted the 8 tests for four genres and two genders. Variables that didn’t show significant differences in any of the 8 tests were excluded. *Creation of models*. Eight models were created using logistic regression (for four genres and two genders). Predictive models included only variables showing significant differences between the depressive and non-depressive group in the M–W test (see step 5).

*Evaluation of models*: The criterion of the quality of each regression model was defined by the Nagelkerke coefficient *r*^2^> 0.4. To assess the predictive power of a model, the following coefficients were calculated: precision (the level of accuracy: the probability that a respondent marked by the model as depressive really is depressive) and recall (also known as sensitivity: the probability that a respondent who really is depressive is classified by the model as depressive). These coefficients are suitable for an unbalanced (unequal) sample (Chawla, 2005). The predictive power of the model was evaluated as sufficient if it met the following conditions: precision >0.8 and recall >0.6. The objectives of the study are exploratory and therefore it is not necessary to use multiple comparison correction (Li et al., 2016). The variables that were inserted into the models went through a two-step selection. Models were further verified according to predefined criteria (precision, recall).

## Results

### Verification of Assumptions and Preparation of Data for Testing and Creating Models

#### Outlier Filtering

In each type of text, 1–4 outliers were detected (see Table 2), a total of 32 texts did not meet the conditions for entering analysis and were automatically discarded from further calculations. Thus, the analyses consisted of 656 texts.

**Table 2:** Frequency of outliers (*N*_resp_ = 172, *N*_text_ = 688).

	Male (*n*_m_*=* 83)		Female (*n_f_* = 89)	
	Non-outliers	Outliers	Non-outliers	Outliers
TXT1	81	2	88	1
TXT2	81	2	85	4
TXT3	81	2	86	3
TXT4	82	1	88	1
*Total*	*325*	*7*	*347*	*9*


#### Reduction of Variables

The stated conditions for variability were fulfilled by 13 quantitative linguistic variables out of the 24 followed ones. These 13 were included in further calculations.

#### Assessment of Normality

As expected, the studied variables were not normally distributed (K–S, sig < 0.05). Due to the non-normal distribution of data, only non-parametric/non-linear procedures were used in further calculations.

#### Lowering Granularity of Depressive Scale

The distribution of scores from the depression subscale of the DASS-21 was the following: men (m_m_ = 5.14, mdn_m_ = 5, min_m_ = 0, max_m_ = 17), women (m_w_ = 4.04, mdn_w_ = 3, min_w_ = 0, max_w_ = 16). Based on their results on the depression subscale, respondents were divided binarily into non-depressive and depressive groups (see Table 3). The cut-off point for these groups (6 points) was derived from the psychometric properties of the test (see Lovibond and Lovibond, 1995). The higher representation of men in the depressive category (about 1/3 of men) compared to women (about 1/5 of women) reflects the characteristics of the research sample (it is unexpected and deserves a separate analysis; let us recall here that the respondents enrolled into the research voluntarily and were included by quota selection. Thus, an identical distribution between men and women and higher prevalence in women were expected.).

**Table 3:** Frequency of depression in sample (*N*_resp_ = 172).

DASS_D	Male (*n*_m_ = 83)	Female (*n_f_* = 89)
Non-depressive (D ≤ 6)	65.5%	81.3%
Depressive (D > 6)	34.5%	18.7%
*Total*	*100*	*100*


### Testing of Intergroup Differences (Depressive vs. Non-depressive)

The depressive and non-depressive groups were compared using a Mann–Whitney U test (M–W test, a non-parametric test for independent samples). The test was conducted separately for men and women as well as for each type of text. Table 4 shows an overview of significances of individual tests (*U* values and mean rank are available from the authors).

**Table 4:** Significance of intergroup differences in ql variables: Mann–Whitney (*N*_resp_ = 172, *N*_text_ = 688).

	Male (*n*_m_ = 83)				Female (*n_f_* = 89)			
	TXT1	TXT2	TXT3	TXT4	TXT1	TXT2	TXT3	TXT4
Singularity index	0.213	0.175	0.503	0.374	0.120	0.653	0.008	0.459
Singularity_P index	0.039	0.208	0.157	0.470	0.211	0.540	0.542	0.291
Negativity index	0.026	0.000	0.013	0.122	0.664	0.613	0.863	0.399
Words per sentence	0.125	0.266	0.062	0.020	0.676	0.475	0.916	0.750
Sentence complexity	0.000	0.030	0.005	0.004	0.518	0.488	0.578	0.721
Punctuations per sentence	0.130	0.826	0.458	0.612	0.290	0.420	0.487	0.009
Coherence index	0.528	0.613	0.732	0.204	0.747	0.236	0.748	0.530
Pronom index	0.059	0.279	0.006	0.205	0.131	0.711	0.210	0.469
Formality index	0.023	0.086	0.001	0.062	0.112	0.566	0.523	0.317
Trager index	0.022	0.011	0.011	0.032	0.128	0.117	0.846	0.189
Readiness_to_action index	0.014	0.049	0.002	0.058	0.171	0.870	0.781	0.965
Aggressiveness index	0.013	0.063	0.008	0.243	0.234	0.468	0.653	0.910
Activity index	0.011	0.025	0.021	0.259	0.076	0.070	0.474	0.390


There was a significant difference in mean rank between the depressive and non-depressive groups for each of the selected linguistic variables (except for the coherence index) in at least one text. The only exception is the coherence index, which according to the result of M–W test does not differentiate between depressive and non-depressive groups in any of the texts, and thus will be excluded from further calculations. Contrary to our expectations, a higher number of significant differences were found among men compared to women, and more often in formal texts (TXT1 and TXT3).

### Creating and Evaluating Models

Eight regression models were created (for four texts among men and four texts among women). Table 5 presents an overview of significance for individual predictors for each model, with each column representing one model.

**Table 5:** Predictors of membership in depressive sample: Logistic regression.

	Male (n*_m_* = 83)				Female (n*_f_* = 89)			
	TXT1	TXT2	TXT3	TXT4	TXT1	TXT2	TXT3	TXT4
Singularity index	0.701	0.062	0.845	0.050	0.058	0.630	0.007	0.349
Singularity_P index	0.044	0.630	0.017	0.585	0.112	0.392	0.746	0.728
Negativity index	0.635	0.534	0.401	0.153	0.169	0.071	0.295	0.247
Words per sentence	0.046	0.172	0.250	0.248	0.046	0.059	0.411	0.194
Sentence complexity	0.004	0.089	0.575	0.536	0.198	0.050	0.618	0.201
Punctuations per sentence	0.236	0.210	0.841	0.087	0.818	0.041	0.677	0.798
Pronom index	0.661	0.042	0.017	0.720	0.385	0.682	0.740	0.323
Formality index	0.479	0.631	0.025	0.723	0.525	0.605	0.234	0.236
Trager index	0.639	0.759	0.019	0.841	0.599	0.139	0.186	0.182
Readiness_to_action index	0.333	0.034	0.017	0.688	0.052	0.941	0.506	0.083
Aggressiveness index	0.162	0.418	0.009	0.863	0.060	0.217	0.999	0.567
Activity index	0.374	0.352	0.007	0.694	0.059	0.554	0.622	0.353
Constant	0.431	0.170	0.059	0.637	0.639	0.248	0.086	0.914


*Each column represents a single model (*N*_*resp*_ = 172, *N*_*text*_ = 688).*

The quality of individual models is described in Table 6 (bold values indicate those model values that meet a predefined quality criterion and allow the model to be accepted).

**Table 6:** Coefficients of model quality and predictive power: Logistic regression (*N*_resp_ = 172, *N*_text_ = 688).

	Male (*n_m_* = 83)				Female (*n_f_* = 89)			
	TXT1	TXT2	TXT3	TXT4	TXT1	TXT2	TXT3	TXT4
Nagelkerke *r*^2^	0.479	0.526	0.435	0.310	0.330	0.670	0.324	0.284
Recall	0.607	0.571	0.556	0.500	0.235	0.800	0.267	0.267
Precision	0.739	0.889	0.789	0.647	0.571	0.889	0.667	0.667


The only model that fulfilled all the defined criteria is the model created on TXT2 (letter from holidays, informal text with positive sentiment) among women. The stated criteria are approximated by three models for men, namely models based on TXT1 (cover letter), 2 (letter from holiday) and 3 (letter of complaint).

## Discussion

The present study focuses on the relationships between linguistic properties of a written text and the level of its writer’s currently experienced depressivity (based on the number of points achieved in the DASS-21 test, participants were divided into depressive and non-depressive group, and these two groups were compared). The chosen methodology is novel: (a) the source for analyses were texts written on an assigned topic under strictly controlled experimental conditions (i.e., not spontaneously written texts), (b) only formal, quantitative linguistic syntactical and morphological variables were subject to analyses (not semantic variables, i.e., only the verbal production, not its content were considered), (c) the research sample was representative of an adult population with respect to age and education (quota selection). We have not come across a study conducted using the same methodological basis. Methodologically similar studies are very scarce (e.g., Litvinova et al., 2016b tried to assess the probability of self-destructive behavior of an individual via formal parameters of their texts).

One of the difficult questions was the choice of linguistic variables to include in the models. We have decided for a statistics-based procedure. In the first step, we have excluded variables with low variability. Sufficient variability has been proven for 6 of 16 selected single morpho-syntactic variables: the number of words per sentence, number of finite verbs per sentence, number of punctuation marks per sentence, proportional variables of relative occurrence of singular, possessive singular, negativity, and for all 8 indexes consisting of combinations and ratios of more morpho-syntactic characteristics: index of coherence, pronominalisation, formality, trager, readiness to action, aggressiveness and activity. This means that, in our study, only a limited amount of selected single morpho-synaptic characteristics was found to be suitable for use in distinguishing between non-depressive and non-depressive texts because of low variability; while all indexes showed sufficient variability. These results can support opinion that it is suitable to use indexes combining in formulas more morpho-syntactic characteristics of a text rather than focus attention on each of the observed linguistic characteristics as an insular unit when looking for relationships between a text and the characteristics of the writer of the text, as some other researchers stated (e.g., Litvinova et al., 2016b).

In step two it was verified discriminatory power of each from these 13 variables via M–W test. The results confirmed that all proposed variables have sufficient discriminatory power to distinguish between texts (always at least one of the texts) of non-depressive and depressive people, except one. The exception is index of coherence. Contrary to our expectations, the present study does not validate the index of coherence (Litvinova et al., 2016b) as a suitable predictor of depression. This index is calculated as the sum of particles plus conjunctions plus prepositions divided by 3 times multiple of number of sentences. We believe that the reason why the index of coherence does not differentiate between non and depressive sample lies in the fact that the index includes the synsemantic parts of speech only. This explanation mirrors Pennebaker’s (2011) argument that personality is most closely related to pronouns and other autosemantic words than synsemantic ones.

Thirteen linguistic variables (6 single morho-syntactic characteristics, 7 indexes combining more morphosyntactic characteristics) were included into the predictive models. Eight predictive models (for 4 different texts and 2 genders) were created and compared with each other. The results show that acceptable level of accuracy show models predicting depression in men sample from texts TXT1 (cover letter), TXT2 (letter from holidays) and TXT3 (complaint), and in women sample from TXT2 (letter from holidays). Across these 4 models, the probability that an individual will be detected as depressive when he/she is not (type II error) is lower than 0.2. The models for men sample show lower quality in criterion recall (their power to detect a depressive individual) than models for women sample. In other words, models built on texts written by men are more likely to fail to detect an individual with depression (type I error) than to erroneously classify an individual as depressive (type II error). Based on these results, it seems justified to state that, pursuant to the morpho-syntactic characteristics of the text, it is more confident to identify depressive women than depressive men.

For explanation, we need to look at gender differences in general and in our study as well. Most current studies show that women experience more depression than men do (e.g., Munce and Stewart, 2007; Klimusová et al., 2016) or the level of depression occurrence is the same for both men and women (Piccinelli and Wilkinson, 2000). However, in our study, men showed a higher level of currently experienced depression than women – it is opposite, unexpected trend. This might be a hit-or-miss feature of our research sample, the unexpected result of self-nomination sampling strategy. Previously diagnosed mental illness has been set as an exclusion criterion for self-nomination into our research no-clinical sample. Because men go to doctors with psychological problems less often than women (e.g., Angst et al., 2002 show that 48% of men and 59% of women with depression seek a doctor), the women with the same intensive depressive symptoms have been visited their doctor and the previous diagnosis made them unable to enter the research as a non-clinical population. It is possible interpretation why there are more depressive men than women in our research sample, even though the prevalence of depression in men is generally lower. However, this circumstance does not explain why predictive model of women sample is stronger than men’s sample predictive models.

The literature has repeatedly described that men and women generally differ in the preference of using some linguistic morpho-syntactic elements in their texts (e.g., Koppel et al., 2002; Argamon et al., 2003; Herring and Paolillo, 2006; Newman et al., 2008; Tausczik and Pennebaker, 2010; Rafi, 2019). Litvinova et al. (2016a) found some text parameters as reliable gender predictors: type-token ratio, formality index, a proportion of prepositions and pronoun-like adjectives, proportion of 100 most frequent words and ration of function (synsemnatic) words to content (autosemantic) words, some of them we operate too. Johannsen et al. (2015) presented a large-scale study of syntactic variation across 11 languages and found that there some universal gender-specific variations across languages: men seem to use numerals and nouns more than women, whereas women use pronouns and verbs more often, men use nominal compounds more often than women. From this point of view, the differences between models found in our study are understandable.

In our study, for women the model predicted depression from TXT2 (letter from holidays: informal text with positive situational sentiment) was of apparently higher quality then models predicting from other texts, while predictive models for men were of comparable quality (with respect to recall and precision metrics) across all the different texts. This result could be related to findings in other research. In this context Biber (1988) stated that women tend to express themselves in the form of “involved” writing while men prefer “informative” writing. Argamon et al. (2003) proved that women tend to present things in a relational way, while men in a non-fictional style of writing. Both cited facts may be related to the fact that the strongest predictive model was found in women just in the text, which is informal – closer to the natural way of verbal presentation of women.

Nor does this knowledge help explain differences found; it provides clear that models predicting the depression differ depending on gender, and that in the future it is necessary to take into account the moderative/mediatory influence of the writer/speaker’s gender in modeling relationships between depression (or other personality characteristics) and text.

Overall, our results indicate that TXT4 (letter of apology) is not a suitable text for creation of a reliable and accurate model predicting depression. On the contrary, TXT2 (letter from holidays) seems to be suitable for creating a good fit predictive model for both men and women.

### Limitations

The present study was conducted on a quota-selected sample of Czech native speakers. Generalization of the findings to Slavonic languages requires further research and generalization to non-Slavonic languages is not recommended. An unexpected limit of this study is the higher percentage of depressive men in the research sample. Due to the relatively small size of the research sample, we did not further verify the results (e.g., by using split half or cross validation).

## Conclusion

The leading motivation for our research is to find ways to use automatic analysis of texts (such as cover letters, letters from holidays, blogs, and comments on social networks) to create predictive models that will reliably detect individuals at risk of a mental disorder (such as depression in the present study) so that they can be provided with help as early as possible. In the present study, we calculated four regression models to predict a higher emotional state of depression. The quality of our models indicates that depression can be predicted from informal text written about a holiday and that the quantitative linguistic characteristics that are most strongly suited to the proposed models for men are the pronominalisation index (the ratio of pronouns to nouns) and readiness to action index (the ratio of verbs to nouns) and for women are sentence complexity (the ratio of finite verbs to number of sentences) and punctuation (the ratio of number of punctuation to number of sentences). We plan to extend our future research to a clinical population to analyze the texts of people with a diagnosed mental disorder, especially with depression or phobias. Given our results and the results of other research (e.g., Rude et al., 2004) we plan to pay more attention to autosemantic words, especially to various types of pronouns.

## Data Availability

The datasets generated and analyzed in this study are available on request from the authors of the article. If interested, please contact the corresponding author.

## Ethics Statement

The project was approved by the Ethics committee of the University of South Bohemia (headed by professor Hana Šantrůčková, president of the Ethics committee of the USB) that confirmed the project was carried out in accordance with the recommendations of the Ethical code of the University of South Bohemia in České Budéjovice. All subjects gave written informed consent in accordance with the Declaration of Helsinki.

## Author Contributions

DK brought the original idea and was the main solver of the grant project. JMH and JH formulated the goals of the study and all authors studied and discussed the relationship between the text and its writer. DK arranged a complete collection of quota sample data. JMH arranged a complete collection of clinical sample data. DK, JH, and JMH designed the online collection, pre-processing, and retention of the data. JMH and PH wrote the introduction. JH designed the data processing procedure, performed all the mathematical and statistical calculations, and described the results. JMH thought out and wrote the discussion and all authors improved the content and formulations of the manuscript. JMH formatted the text and DK ensured professional proofreading and uploading of the manuscript for the review process.

## Conflict of Interest Statement

The authors declare that the research was conducted in the absence of any commercial or financial relationships that could be construed as a potential conflict of interest.
